# Comparison of the efficacy and safety of a 730‐nm picosecond titanium sapphire laser and a 1064‐nm picosecond neodymium yttrium aluminum garnet laser for the treatment of acquired bilateral nevus of Ota‐like macules: A split‐face, evaluator‐blinded, randomized, and controlled pilot trial

**DOI:** 10.1111/jocd.16511

**Published:** 2024-08-24

**Authors:** Wanxin Chen, Zhongshuai Wang, Zhenzhen Li, Chen Yuan, Xiaofeng Zhang, Li Li, Yan Yan, Baoxi Wang

**Affiliations:** ^1^ Department of Dermatology Plastic Surgery Hospital, Chinese Academy of Medical Sciences and Peking Union Medical College Beijing China

**Keywords:** acquired bilateral nevus of Ota‐like macules, Hori's nevus, picosecond laser, titanium sapphire laser

## Abstract

**Background:**

The picosecond neodymium yttrium aluminum garnet laser (PNYL) has been successfully used in treating acquired bilateral nevus of Ota‐like macules (ABNOM). The 730‐nm picosecond titanium sapphire laser (PTSL) is an emerging tool for pigmentary disorders. However, no studies have compared two different wavelengths of picosecond laser for the treatment of ABNOM.

**Aims:**

To compare the efficacy and safety of the 730‐nm PTSL with the 1064‐nm PNYL in the treatment of ABNOM.

**Methods:**

Fifteen participants with ABNOM were randomized to undergo a single session of either the 730‐nm PTSL on one side of the face and 1064‐nm PNYL on the other side. Efficacy and safety assessments were performed by blinded visual evaluations at baseline, 12 weeks, and 24 weeks posttreatment. Participants' satisfaction and adverse effects were recorded.

**Results:**

Compared to baseline, The 730‐nm PTSL‐treated side showed better improvement than that of the 1064‐nm PNYL‐treated side at 24 weeks posttreatment (1.67 ± 1.047 vs. 0.87 ± 0.640, *p* = 0.027). There were no significant differences in pain sensation and participants' satisfaction between the two laser treatments.

**Conclusions:**

The 730‐nm PTSL is more effective than the 1064‐nm PNYL in the treatment of ABNOM.

## 1 INTRODUCTION

Acquired bilateral nevus of Ota‐like macules (ABNOM) or Hori's nevus was first documented by Hori et al. in 1984.^1^ ABNOMs represent a prevalent dermal pigmentation disorder, predominantly affecting middle‐aged women in Eastern Asia, with a documented prevalence of 2.5% in Shanghai, China.^2^ Histologically, ABNOM is characterized by the predominant distribution of melanocytes in the upper to mid‐dermis.^3^ Laser therapy is considered the optimal intervention for ABNOM, with various laser types being used, including alexandrite laser, neodymium‐doped yttrium aluminum garnet (Nd:YAG) laser, erbium‐doped yttrium aluminum garnet (Er:YAG) laser, ruby laser, and CO_2_ laser.^4^ Recent investigations have highlighted the superiority of picosecond lasers over nanosecond lasers in treating pigmented disorders. Recent investigations have highlighted the superiority of picosecond lasers over nanosecond lasers in treating pigmented disorders.^5^

Despite the recognized advantages of picosecond laser technology, it is important to note that varying laser wavelengths may exhibit different efficacy levels in addressing ABNOM due to their distinct penetration depths. A systematic review and meta‐analysis compared the efficacy of different wavelengths of Q‐switched lasers in treating nevus of Ota. The results demonstrated that the 755‐nm Q‐switched alexandrite laser had a superior pigment clearance rate compared to the 1064‐nm Nd:YAG laser (48.3% vs. 41%).^6^ A randomized controlled study on 86 cases of freckles compared the efficacy of a 730‐nm picosecond laser with a 755‐nm picosecond laser, revealing similar effects but less pain with the 730‐nm wavelength.^7^ Limited case reports have shown comparable efficacy of 730‐ and 785‐nm picosecond lasers in treating nevus of Ota.^8^ One prospective study found that 76.7% of patients achieved 76%–100% lesion improvement after three treatments with a 755‐nm picosecond laser.^5^ In contrast, a retrospective study indicated that 85.7% of patients achieved more than 50% lesion clearance after five treatments with a 1064‐nm picosecond laser, despite both wavelengths target melanin.^9^ Each wavelength has its advantages: The 730‐nm picosecond titanium sapphire laser (PTSL) is proficient in targeting melanin and melanocytes, while the 1064‐nm picosecond neodymium‐doped yttrium aluminum garnet laser (PNYL) penetrates deeper tissues.

Notably, there is a conspicuous absence of comprehensive, prospective comparative investigations comparing the efficacy and safety of the 730‐nm PTSL against the 1064 nm PNYL in treating ABNOM. Our study represents a pioneering effort, employing a prospective, split‐face, evaluator‐blinded, randomized, and controlled design to systematically assess the efficacy and safety of these two laser modalities in the context of ABNOM treatment.

## 2 MATERIALS AND METHODS

### 2.1 Study design

This prospective, randomized, evaluator‐blinded, split‐face, pilot study was conducted from March 2023 to September 2023 in the Department of Dermatology at a leading hospital in Beijing, China. Ethical approval for the study was obtained from the Investigational Review Board of the same institution (2022[235]). Prior to study inclusion, all enrolled patients provided both written and verbal informed consents in accordance with established ethical protocols.

### 2.2 Patient selection

Participants aged 18–65 years with bilaterally distributed facial lesions were included in this comprehensive study. Exclusion criteria included individuals who had undergone laser treatment, energy‐based interventions, cryotherapy, esthetic surgery, or chemical peels within the past 24 weeks. Also excluded were those with coexisting hyperpigmented dermatoses, active infections, heightened light sensitivity, recent use of bleaching agents (within the past 4 weeks), or a history of hypertrophic scars/keloids. Additionally, individuals with severe systemic or psychiatric disorders, as well as pregnant or lactating women, were deemed ineligible for participation. The diagnosis of ABNOM was confirmed by a professional dermatologist through a thorough assessment of historical information and lesion characteristics. Demographic details, including sex, age, and Fitzpatrick skin type, were recorded for each participant. Fitzpatrick skin type was determined based on responses to a sun‐exposure reaction questionnaire or objectively assessed by the investigator. Comprehensive photographic documentation was conducted prior to the initial laser treatment and during subsequent follow‐up visits. Prior to commencement of the study, participants were provided with both written and verbal consent forms, detailing the study protocols, alternative treatments, potential benefits, adverse effects, and corresponding measures.

### 2.3 Laser treatments

**FIGURE 1:**
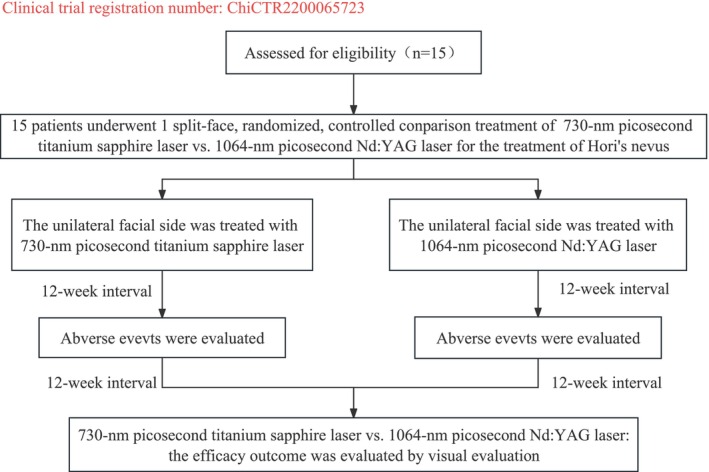
The trial profile.

For each case, the selection of treating areas on the left or right side of the face was determined through a random allocation process. The options were either 730‐nm, 250‐ps titanium sapphire laser (PicoWay; Syneron‐Candela) or 1064‐nm, 450‐ps Nd:YAG laser (PicoWay; Syneron‐Candela). The randomization procedure was carried by a dermatologist not involved in the trial, using random sequence software to generate a randomized number sequence (http://www.dxy.cn/bbs/topic/21117904). Each patient was assigned a number: Those with odd numbers received treatment with the 730‐nm PTSL on the left side of the face, while those with even numbers received treatment with the 1064‐nm PNYL on the right side. (Clinical trial registration number: ChiCTR2200065723) (Figure 1). Test spots were performed to determine an appropriate energy level for each patient, establishing treatment end points defined as immediate whitening followed by redness and edema for the 730‐nm PTSL or delayed punctuate purpura for the 1064‐nm PNYL.

The treatment parameters for the 730‐nm group included a 2 mm spot size, 2.25–4 J/cm^2^ fluence, and a single pass without overlapping. For the 1064‐nm group, the parameters were a 3 mm spot size, 2.2–3.7 J/cm^2^ fluence, and a single pass without overlapping. To minimize treatment energy bias, the same laser operator performed treatments on both sides of the face, ensuring a consistent treatment end point response. After treatment, the treated areas were cooled using cold packs for 15–30 min. Patients were instructed to apply 0.1% Hydrocortisone Butyrate Cream to the treated areas twice a day for 3 days and to follow strict sun protection measures. Follow‐up evaluations was conducted at 12 and 24 weeks posttreatment.

### 2.4 Efficacy and safety evaluation

Standard digital photographs were acquired using Think View (CBS, Taiwan, China) both pretreatment and at 12 and 24 weeks posttreatment. The primary clinical outcome evaluation was conducted by two experienced dermatologists who were blinded to the study conditions at baseline and during all follow‐up assessments. The assessment focused on the percentage of lesion improvement, categorized as follows: perfect (76%–100%, score 4), excellent (51%–75%, score 3), good (26%–50%, score 2), fair (1%–25%, score 1), and no improvement (0%, score 0). In cases where different treatment scores were assigned to a particular photograph, the mean score was adopted.

Upon completion of the study, patients were asked to provide feedback on their satisfaction levels using a Likert Satisfaction Scale, rated as very dissatisfied (0), dissatisfied (1), slightly satisfied (2), satisfied (3), or very satisfied (4). No topical anesthetics were utilized before treatment. Patients were requested to indicate their level of pain immediately following the 730‐nm PTSL or 1064‐nm PNYL treatments using a visual analog scale (VAS), ranging from 0 (no pain at all) to 10 (worst pain ever).

Transient and permanent adverse effects associated with laser treatments, including swelling, crusting, blistering, bleeding, post‐inflammatory hyperpigmentation (PIH), hypopigmentation (PIHo), and scaring, were evaluated by two investigators who were blinded to the treatment allocation. Any inquiries regarding side effects were addressed through phone calls, and patients provided self‐portraits via social media applications.

### 2.5 Data analysis

The collected data were analyzed using SPSS 20.0 (IBM, Armonk, NY, USA). Numerical data were expressed as mean and standard deviation, while qualitative data were expressed as percentages. The Shapiro–Wilk test was employed to assess the normality of quantitative data. Nonparametric tests were utilized for data that were not normally distributed. The Mann–Whitney test was conducted to evaluate differences in pain, lesion improvement, and participant satisfaction levels between the two laser treatments. All tests were two‐tailed, and statistical significance was defined as *p* < 0.05.

## 3 RESULTS

Fifteen patients (comprising 1 male and 14 females) successfully completed the entire study. The mean age of the participants was 28.60 years, with an age range between 23 and 40 years. Fitzpatrick skin types III and IV were identified in 53.3% (8/15) and 40.0% (6/15) of the patients, respectively. Quantitative evaluation indicators are detailed in Table 1.

**TABLE 1:** Quantitative outcomes of the two laser treatment groups.

Variables	730‐nm PTSL	1064‐nm PNYL	*p*
Quartile improvement scale	1.67 ± 1.047	0.87 ± 0.640	0.027
Likert satisfaction scale	2.40 ± 1.121	1.73 ± 0.799	0.134
VAS	6.27 ± 1.534	5.60 ± 1.639	0.330

Note: All values are expressed as mean ± SD or percentage.

Abbreviations: PNYL, picosecond neodymium yttrium aluminum garnet laser; PTSL, picosecond titanium sapphire laser; VAS, visual analog scale.

### 3.1 Efficacy outcomes

**FIGURE 2:**
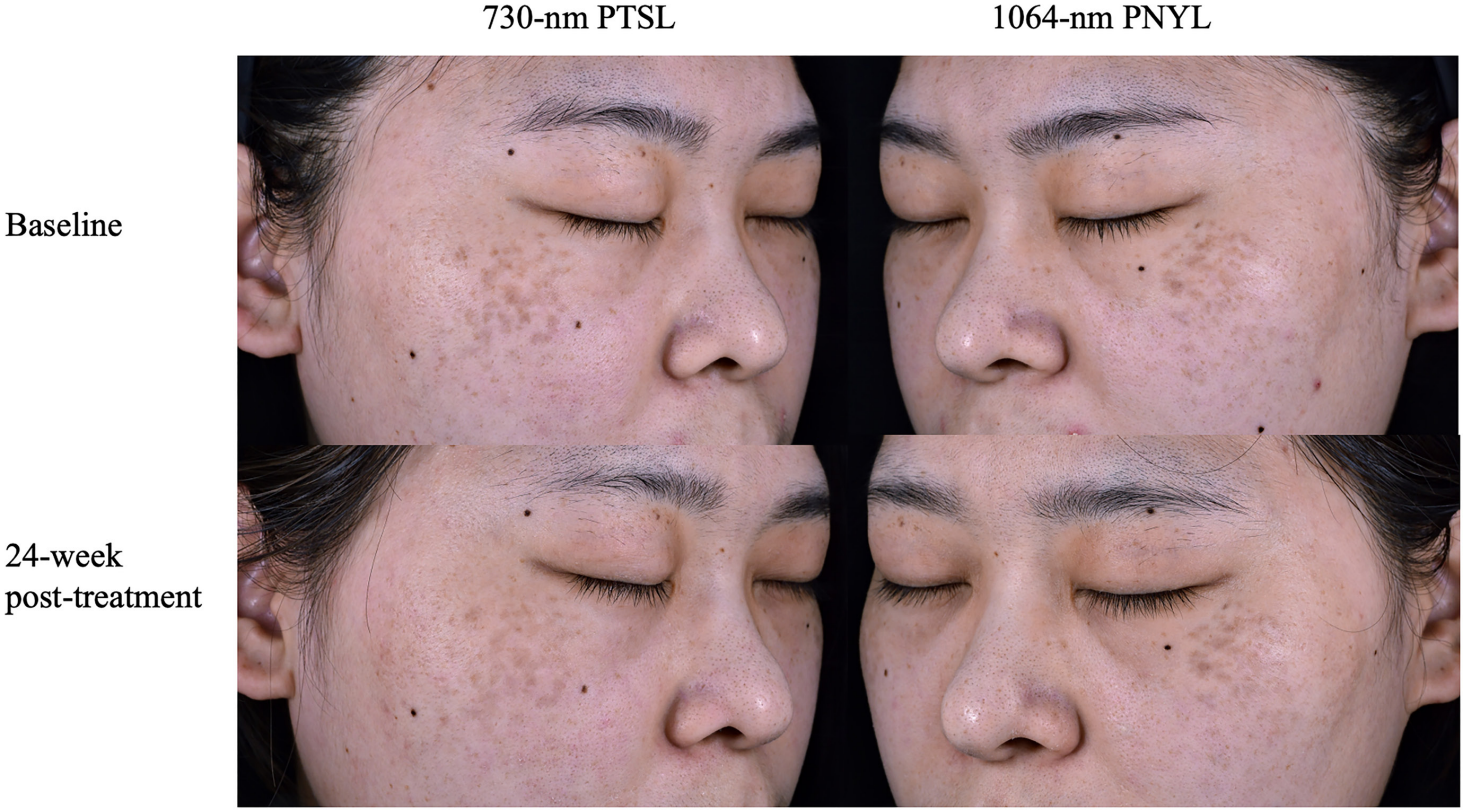
Before and after photographs of a 27‐year‐old female with Fitzpatrick skin phototype III. Right side of face was treated with 730‐nm PTSL treatment (2 mm, 3.0 J/cm^2^); left side of face was treated with 1064‐nm PNYL treatment (3 mm, 3.1 J/cm^2^). PTSL, picosecond titanium sapphire laser; PNYL, picosecond neodymium yttrium aluminum garnet laser.

**FIGURE 3:**
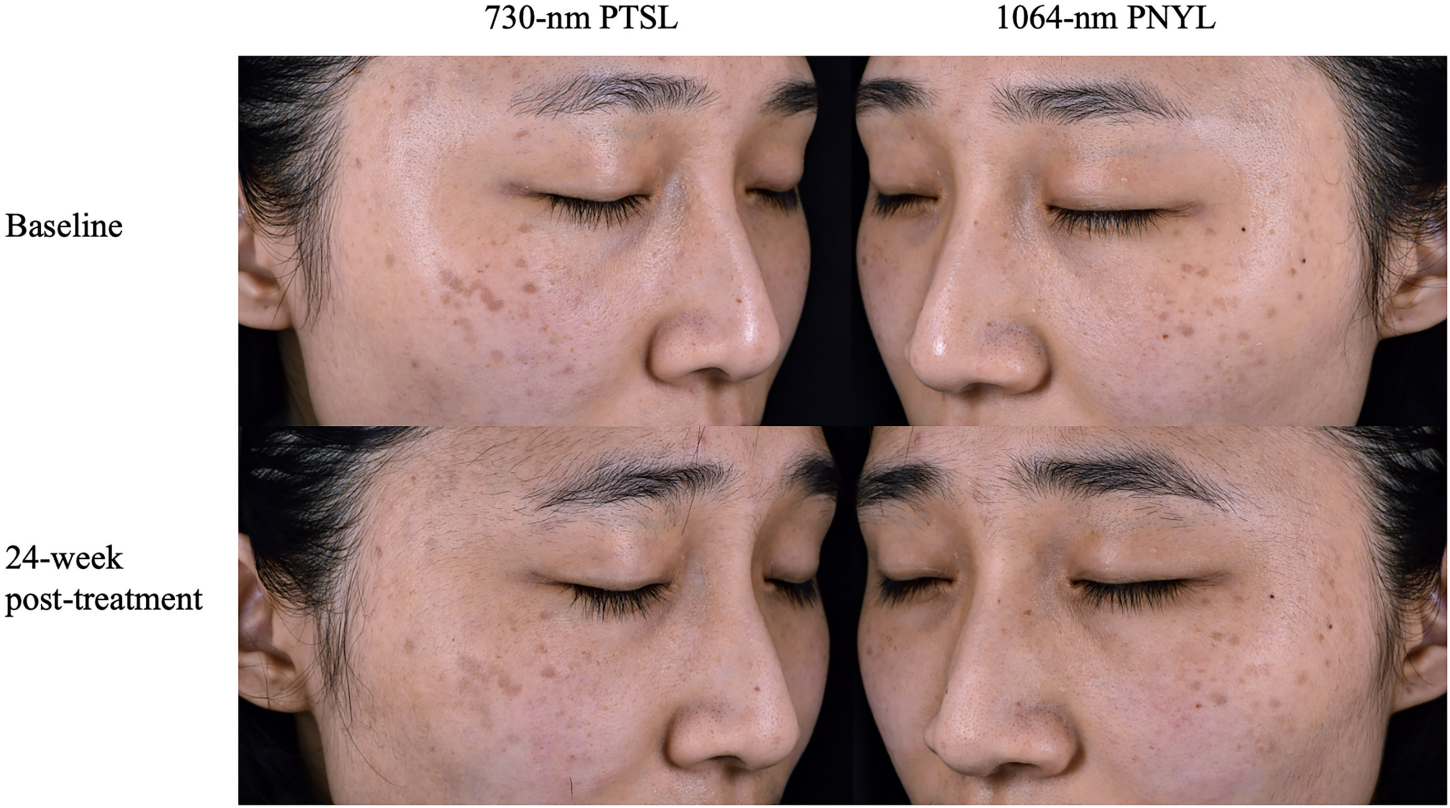
Before and after photographs of a 28‐year‐old female with Fitzpatrick skin phototype III. Right side of face was treated with 730‐nm PTSL treatment (2 mm, 2.25 J/cm^2^); left side of face was treated with 1064‐nm PNYL treatment (3 mm, 2.2 J/cm^2^). PTSL, picosecond titanium sapphire laser; PNYL, picosecond neodymium yttrium aluminum garnet laser.

At the 24‐week follow‐up, the 730‐nm PTSL arm exhibited statistically superior performance compared to the 1064‐nm PNYL arm among the 15 patients (1.67 ± 1.047 vs. 0.87 ± 0.640, *p* = 0.027). No patient achieved complete lesion clearance (Tables 1 and 2). Figures 2 and 3 visually depict the pre‐ and posttreatment photographs of two patients. Additionally, there were no significant differences in participant satisfaction between the two arms (2.40 ± 1.121 vs. 1.73 ± 0.799, *p* = 0.134; Tables 1 and 3).

**TABLE 2:** Lesion improvement scores of the two laser treatment groups.

Quartile improvement scale	No./total no. of participants	
	730‐nm PTSL	1064‐nm PNYL
4 = perfect (76%–100%)	0/15	0/15
3 = excellent (51%–75%)	3/15	0/15
2 = good (26%–50%)	4/15	2/15
1 = fair (1%–25%)	5/15	9/15
0 = no improvement (0%)	2/15	4/15

Abbreviations: PNYL, picosecond neodymium yttrium aluminum garnet laser; PTSL, picosecond titanium sapphire laser.

**TABLE 3:** Participants satisfaction rates of the two laser treatment groups.

Likert satisfaction scale	No./total no. of participants	
	730‐nm PTSL	1064‐nm PNYL
4 = very satisfied	4/15	0/15
3 = satisfied	1/15	2/15
2 = slightly satisfied	7/15	8/15
1 = dissatisfied	3/15	4/15
0 = very dissatisfied	0/15	1/15

Abbreviations: PNYL, picosecond neodymium yttrium aluminum garnet laser; PTSL, picosecond titanium sapphire laser.

### 3.2 Adverse effects

According to the VAS scores, there was no statistically significant difference in the average pain sensation between the 730‐nm PTSL arm and the 1064‐nm PNYL arm (6.27 ± 1.534 vs. 5.60 ± 1.639, *p* = 0.330; Table 1). Most patients experienced mild‐to‐moderate erythema and edema after treatments. The edema resolved within 24 h and the erythema subsided completely within 1 week.

The PIH rates in the 730‐nm group were slightly higher than those in 1064‐nm group at the 12‐week (26.67% vs. 20.00%). However, at the 24‐week follow‐up, the PIH rates were identical between the two groups (20% vs. 20%). Two out of 15 participants continued to develop PIH on both sides of face in each group at both follow‐up visits. Notably, one participant in the 1064‐nm laser arm did not develop PIH until the second follow‐up visit. Additionally, one participant developed delayed PIH on both sides of face in each group at the 24‐week mark and one participant experienced worsening melasma beyond the treated area bilaterally. Importantly, none of the patients reported bleeding, scarring, blistering, scabbing, or PIHo.

## 4 DISCUSSION

ABNOM represents a prevalent dermal pigmented condition predominantly affecting young Asian women. Characteristic features include blue‐brown to slate‐gray macules, mainly located on the bilateral malar region, exhibiting clear boundaries without fusion. The advent of Q‐switched lasers in the 1990s significantly alleviated this condition, with reported clearance rates ranging from 51% to 100% in 92.3% of patients after five or more treatment sessions using the 1064‐nm Q‐switched laser.^10^, ^11^ However, since the introduction of the first picosecond laser in 2012, notable advantages in treating ABNOM have been observed.^12^ Ding et al. conducted a review of 225 Chinese patients treated with the 755‐nm alexandrite laser for ABNOM, revealing that 60% of patients achieved a clinical clearance rate exceeding 90% after four laser treatments.^13^ The treatment efficacy demonstrated a positive correlation with the number of treatments. In a 2‐year prospective randomized controlled study by Yu et al., a direct comparison highlighted significant differences in the efficacy between picosecond and nanosecond lasers for ABNOM. A clearance rate of 76%–100% was observed in 76.7% of patients treated with the 755‐nm alexandrite laser three times every 6 months. Importantly, the efficacy of the 755‐nm alexandrite laser surpassed that of the 755‐nm Q‐switched laser.^5^ Similarly, Ungaksornpairote et al. established the superiority of the 1064‐nm picosecond laser over the 1064‐nm ns laser in the treatment of ABNOM.^14^

The 730‐nm QTSL represents a novel wavelength of picosecond laser, generated through the conversion of the 532‐nm s harmonic of the primary 1064‐nm ps‐laser into 730‐nm laser light, employing a laser‐pumped handpiece.^15^ This laser variant boasts the shortest pulse duration of 250 ps among contemporary picosecond lasers, theoretically resulting in more potent photomechanical effects and fewer associated side effects.^16^ In a study conducted in 2020, Bernstein et al. investigated the application of the 730‐nm picosecond laser in tattoo removal and observed favorable outcomes for green, blue, and purple tattoos, with an average clearance rate ranging from 77% to 83%.^17^ Subsequently, Lee et al. reported the initial success of applying a 730‐nm QTSL to Asian patients with freckles and lentigines.^15^ Kauvar et al. presented findings from a study involving 20 patients with facial and non‐facial lentigines treated with a 730‐nm picosecond laser, revealing that 74% of patients achieved more than 50% lesion clearance by Week 12.^18^ Histological analysis confirmed the minimal tissue damage associated with the short pulse duration laser.

In a two‐center, randomized controlled trial by Zhang et al., comparing the efficacy of 730‐nm and 755‐nm picosecond lasers in 86 patients with freckles, results indicated a high lesion clearance rate in the 730‐nm picosecond laser group (68.99 ± 7.42%), with no significant difference compared to the 755‐nm picosecond laser group (69.27 ± 7.75%). Notably, the pain experienced by the former group was significantly lower than that of the latter (*p* < 0.0001).^7^ Additionally, Loh et al. explored the application of the 730‐nm picosecond laser in nevus of Ota, demonstrating that four sessions at 1‐month intervals achieved a lesion clearance rate exceeding 75%.^8^ This suggests that the 730‐nm picosecond laser holds substantial potential for the treatment of ABNOM, a condition histologically similar to nevus of Ota.

We conducted a prospective, split‐face, evaluator‐blinded, randomized, and controlled pilot study to assess the efficacy of a 730‐nm PTSL compared to a 1064 nm PNYL in Asian patients with ABNOM. To the best of our knowledge, this study marks the initial investigation into the effectiveness of a 730‐nm PTSL specifically for Asian patients with ABNOM.

The efficacy of the 730‐nm PTSL demonstrated significant superiority over the 1064‐nm PNYL. This discrepancy may be attributed to the higher melanin‐hemoglobin absorption coefficient ratio at 730 nm, which surpasses that at 1064 nm.^8^ Consequently, the superficial dermal blood vessels of ABNOM experienced less competitive absorption of the laser, allowing the laser to maximize its impact on melanin. Additionally, the 730‐nm laser's shorter pulse duration contributed to a more robust photomechanical effect, facilitating the fragmentation of pigment particles into smaller entities and thereby promoting effective tissue clearance.

In this investigation, patients in both groups reported moderate levels of pain, and the outcomes revealed no statistically significant difference in pain sensation between the two arms. Such pain levels were slightly higher than those experienced with a 755‐nm picosecond laser in treating ABNOM.^5^ PIH following laser surgery is a common occurrence in the Asian population. The reported incidence of PIH in ABNOM treated with picosecond laser and Q‐switched laser ranges from 27.77% to 64.3% and from 54% to 94.9%, respectively.^5^, ^14^ Notably, the incidence of PIH with picosecond laser in our study is lower than previous studies. At Week 12, the 730‐nm group had slightly higher PIH rates than the 1064 group, which was compatible at Week 24. One participant experienced worsening melasma beyond the treated area bilaterally, although the lesions had improved. Additionally, two participants, who were both around 40 years old, experienced persistent PIH at follow‐up visits, which may be related to slow pigment metabolism. This suggests the need for extended follow‐up durations and thorough counseling with the patients before treatments. It is noteworthy that none of the participants in our study exhibited PIHo, blistering, bleeding, or scarring.

Limitations of our study include a comparatively small sample size, single‐blinding, the absence of objective indicators for efficacy evaluation, a restricted number of administered treatments, and limited follow‐up durations. Given the small sample size, the potential risk of Type II errors cannot be neglected. To address this, we performed a post hoc power analysis and found an actual statistical power of 0.67 (typically 80% or higher is desired). The power of study warrants further strengthening.

As a pilot study, we aim to lay the groundwork for future research. Considering the practical challenges of blinding in the treatments with different wavelengths of lasers, only the two evaluators were completely blinded to the randomization outcome and the treatment parameters the lasers. Although we used standard photography to ensure consistent light source and photo resolution, the subjective evaluation of the evaluators may introduce some bias into the study.

We did not achieve complete lesion clearance because of the single treatment. The 24‐week follow‐up period may not be sufficient to document all side effects comprehensively and an extended follow‐up period may provide valuable insights for both clinical practice and future research directions. Prospective research endeavors should aim to address these limitations through multicenter randomized controlled trials with substantial sample sizes. Such endeavors would be instrumental in delineating the optimal parameters for the application of the 730‐nm picosecond laser in treating ABNOM.

In summary, the 730‐nm PTSL is more effective than the 1064‐nm PNYL in treating ABNOM.

## AUTHOR CONTRIBUTIONS

Wanxin Chen designed and performed the study (participant recruitment and screening, photography, data collection and interpretation, and statistical analysis), discussed the results, and wrote the manuscript. Zhongshuai Wang performed randomization procedure and approved the manuscript. Zhenzhen Li performed the study (clinical assessment) and approved the manuscript. Chen Yuan performed the study (clinical assessment) and approved the manuscript. Xiaofeng Zhang performed the study (laser treatment) and approved the manuscript. LiLi performed the study (laser treatment) and approved the manuscript. Yan helped supervise the project, discussed the results, and approved the manuscript. Baoxi Wang helped design the study, supervised the project, discussed the results, revised and approved the manuscript.

## CONFLICT OF INTEREST STATEMENT

The authors declare no conflict of interests.

## ETHICS STATEMENT

Ethical approval for the study was obtained from the Investigational Review Board of the Hospital of Plastic Surgery, Chinese Academy of Medical Sciences (2022[235]). Both written and verbal informed consents were obtained in accordance with established ethical protocols from all participants. All participants provided photography consents.

## Supporting information


Table S1.

## Data Availability

The data that support the findings of this study are available from the corresponding author upon reasonable request.
