# Supplementary material for: Comparison of the efficacy and safety of a 730‐nm picosecond titanium sapphire laser and a 1064‐nm picosecond neodymium yttrium aluminum garnet laser for the treatment of acquired bilateral nevus of Ota‐like macules: A split‐face, evaluator‐blinded, randomized, and controlled pilot trial
Source: J Cosmet Dermatol. 2024 Aug 24;23(12):3961–7. doi: 10.1111/jocd.16511 (PMC11626365; doi:10.1111/jocd.16511)
Supplement: Supplementary file 1 — Table S1. [file JOCD-23--s001.doc]

**Table S1 Demographic Information**

| **Number** | **Gender** | **Age** | **Ethnicity** | **Fitzpatrick skin type** | **730nm** | **1064nm** |
| --- | --- | --- | --- | --- | --- | --- |
|  |  |  |  |  | **Fluence** | **Fluence** |
| 001 | Female | 23 | Asian | 4 | 2.5 | 2.8 |
| 002 | Female | 28 | Asian | 3 | 2.25 | 2.2 |
| 003 | Female | 26 | Asian | 3 | 2.25 | 2.5 |
| 004 | Female | 28 | Asian | 3 | 2.5 | 2.5 |
| 005 | Female | 40 | Asian | 3 | 2.25 | 2.8 |
| 006 | Female | 24 | Asian | 3 | 2.75 | 2.8 |
| 007 | Female | 26 | Asian | 4 | 2.25 | 2.8 |
| 008 | Female | 24 | Asian | 4 | 2.5 | 3.1 |
| 009 | Male | 24 | Asian | 4 | 2.5 | 3.7 |
| 010 | Female | 31 | Asian | 3 | 3 | 3.1 |
| 011 | Female | 26 | Asian | 4 | 2.75 | 2.8 |
| 012 | Female | 27 | Asian | 3 | 2.5 | 2.5 |
| 013 | Female | 36 | Asian | 4 | 2.25 | 2.8 |
| 014 | Female | 36 | Asian | 5 | 2.5 | 2.8 |
| 015 | Female | 30 | Asian | 3 | 4 | 3.4 |

Fluence：J/cm^2^
